# Programming of subthalamic nucleus deep brain stimulation with hyperdirect pathway and corticospinal tract‐guided parameter suggestions

**DOI:** 10.1002/hbm.26390

**Published:** 2023-06-15

**Authors:** Alba Segura‐Amil, Andreas Nowacki, Ines Debove, Katrin Petermann, Gerd Tinkhauser, Paul Krack, Claudio Pollo, T. A. Khoa Nguyen

**Affiliations:** ^1^ Department of Neurosurgery University Hospital Bern Bern Switzerland; ^2^ ARTORG Center for Biomedical Engineering Research University Bern Bern Switzerland; ^3^ Department of Neurology University Hospital Bern Bern Switzerland

**Keywords:** deep brain stimulation, hyperdirect pathway, Parkinson's disease, programming, tractography

## Abstract

Deep brain stimulation (DBS) of the subthalamic nucleus (STN) is an effective treatment for advanced Parkinson's disease. Stimulation of the hyperdirect pathway (HDP) may mediate the beneficial effects, whereas stimulation of the corticospinal tract (CST) mediates capsular side effects. The study's objective was to suggest stimulation parameters based on the activation of the HDP and CST. This retrospective study included 20 Parkinson's disease patients with bilateral STN DBS. Patient‐specific whole‐brain probabilistic tractography was performed to extract the HDP and CST. Stimulation parameters from monopolar reviews were used to estimate volumes of tissue activated and to determine the streamlines of the pathways inside these volumes. The activated streamlines were related to the clinical observations. Two models were computed, one for the HDP to estimate effect thresholds and one for the CST to estimate capsular side effect thresholds. In a leave‐one‐subject‐out cross‐validation, the models were used to suggest stimulation parameters. The models indicated an activation of 50% of the HDP at effect threshold, and 4% of the CST at capsular side effect threshold. The suggestions for best and worst levels were significantly better than random suggestions. Finally, we compared the suggested stimulation thresholds with those from the monopolar reviews. The median suggestion errors for the effect threshold and side effect threshold were 1 and 1.5 mA, respectively. Our stimulation models of the HDP and CST suggested STN DBS settings. Prospective clinical studies are warranted to optimize tract‐guided DBS programming. Together with other modalities, these may allow for assisted STN DBS programming.

## Graphical abstract

This retrospective study included 20 Parkinson's disease patients with STN DBS. Patient‐specific tractography was performed to compute stimulation models of the hyperdirect pathway and corticospinal tract. Both models were used to suggest stimulation parameters for directional DBS leads.

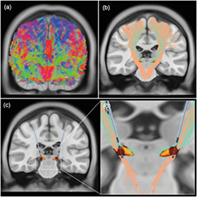

**Abbreviations**
BA: Brodmann Areas
CST: corticospinal tract
DBS: deep brain stimulation
dMRI: diffusion magnetic resonance imaging
HDP: hyperdirect pathway
MNI: Montreal Neurological Institute template space
SIFT2: spherical‐deconvolution informed filtering of tractograms
STN: subthalamic nucleus
TE: echo time
TR: repetition time
VTA: volume of tissue activated

## 1 INTRODUCTION

Deep brain stimulation (DBS) is an effective treatment for advanced Parkinson's disease patients to control motor symptoms such as bradykinesia, rigidity, or tremor. The subthalamic nucleus (STN) is a common DBS target in Parkinson's disease as it plays a relevant role in motor control via the direct, indirect, and hyperdirect pathways (HDP) (Emmi et al., 2020). Even if the mechanisms of action of STN DBS are not fully understood, activation of the HDP has been shown to contribute to the therapeutic effect (Miocinovic et al., 2018; Oswal et al., 2021).

Stimulation models of the HDP and other pathways have been previously developed to study the mechanisms of effect of DBS. These models could also be used to suggest stimulation parameters. They require tractography to identify the pathways and modeling of electrical stimulation to estimate the activation of these pathways. Then, the models' suggestions can be compared with the clinical parameters to evaluate their accuracy and usefulness to facilitate post‐operative programming (Jaradat et al., 2022; Krack et al., 2002; Mahlknecht et al., 2017). Methods to quantitatively estimate the pathway activation are still evolving and are not fully established. Previous studies estimated pathway activation through streamline counts, that is, the number of streamlines activated (Brunenberg et al., 2012; Plantinga et al., 2016; Vanegas‐Arroyave et al., 2016). But these counts have limitations and may not be a good indicator of the biological density of axons (Jones et al., 2013; Smith et al., 2015). One approach to better estimate pathway activation is through filtering algorithms such as SIFT (Smith et al., 2013, 2015) or COMMIT (Schiavi et al., 2020), which assign *weights* to streamlines.

The main aim of the current study was to retrospectively suggest stimulation parameters based on the stimulation models for the HDP and corticospinal tract (CST). We developed two models with weighted streamlines: one for the HDP to estimate the therapeutic effect threshold and another for the CST to estimate the capsular side effect threshold. We used the models to suggest stimulation parameters and compared these against the monopolar reviews. These reviews are currently standard‐of‐care in DBS programming and can be time‐consuming and complex with modern directional DBS systems that have eight contacts or more. Computer‐assisted programming with tract‐guided suggestions may therefore in the future facilitate programming and improve patient care.

## 2 MATERIALS AND METHODS

### 2.1 Patients

This monocentric retrospective study included 20 Parkinson's disease patients (10 females, age range: 35–81 years) who underwent bilateral implantation of the STN between August 2018 and June 2020. The inclusion criteria were the following: (1) signed general consent (study approved by local ethics committee [2020‐02392]); (2) patients implanted with Boston Vercise Cartesia directional leads (Boston Scientific, Marlborough, MA); (3) available preoperative diffusion MRI (dMRI) imaging.

### 2.2 Image acquisition

Imaging was performed on a 3 Tesla scanner (Magnetom Skyra Fit, Siemens, Germany) using a 32‐channel receive head coil. The preoperative protocol included a T1‐weigthed MPRAGE (TR = 2020 ms, TE = 3.49 ms, voxel resolution 1 × 1 × 1 mm^3^, acquisition time 4 min), a T2‐weighted sequence (TR = 2400 ms, TE = 225 ms, voxel resolution 1 × 1 × 1 mm^3^, acquisition time 14 min), and a dMRI sequence acquired in the Siemens q‐space mode (TR = 5900 ms, TE = 111 ms, voxel resolution 2.2 × 2.2 × 2.2 mm^3^, in‐plane acceleration GRAPPA factor of 2, partial Fourier 7/8, field of view 211 × 211 mm^2^, acquisition time 12 min). Diffusion weighting, with multiple b‐values in the range of 0–3000 s/mm^2^ was applied along 123 directions uniformly distributed on a sphere (Supporting Information Table S1). A postoperative CT scan was also acquired.

### 2.3 Surgical procedure and postoperative assessment

For a detailed description of our targeting approach and operative procedure, we refer to a previously published report (Nowacki et al., 2018). All patients underwent a monopolar contact review off dopaminergic medication after 4–6 months to determine the therapeutic effect of DBS (Nguyen et al., 2019). The tested contacts included the omnidirectional contacts, the directional contacts, as well as pseudoring stimulation, that is, activating the three directional contacts on the same level together. The stimulation amplitude was increased from 1 to 8 mA in 0.5 mA steps (unless persistent side effects appeared beforehand), while frequency and pulse width were kept constant at 130 Hz and 60 μs. The effect threshold was defined as the lowest current amplitude that resulted in full, or maximal, reduction of wrist rigidity. The side effect threshold was defined as the lowest current amplitude that resulted in persistent capsular side effects. Data from 40 leads were included, with effect thresholds for 275 contacts and side effect thresholds for 281 contacts (Supporting Information Table S2).

Lead localization was performed with the Lead‐DBS toolbox (version 2.5) (Horn & Kühn, 2015) in Matlab 2019b (The Mathworks, Natick, MA) as in (Nguyen et al., 2019). For each lead and contact, VTAs were computed as thresholded e‐fields in the range of 1–8 mA with steps of 0.5 mA. The SimBio/Fieldtrip pipeline was used with default values for grey and white matter conductivities (0.33 and 0.14 S/m) and a threshold of 0.2 V/m to obtain binarized VTAs in MNI template space (Montreal Neurological Institute). All VTAs were then warped to patient‐specific dMRI space with the inverse normalization, which was computed with Advanced Normalization Tools in Lead‐DBS.

### 2.4 Tractography

#### 2.4.1 Diffusion MRI pre‐processing

Prior to tractogram generation, preliminary denoising (Veraart et al., 2016) and removal of Gibb's ringing artefacts (Kellner et al., 2016) were performed in MRtrix3 (version 3.0) (Tournier et al., 2019). FMRIB's Software Library (FSL, version 6.0.3) (Jenkinson et al., 2012) was used for the correction of geometric distortion and eddy current artefacts. Finally, correction for motion and eddy currents (Andersson & Sotiropoulos, 2016) and susceptibility induced distortions (Andersson et al., 2003) was performed. As no reversed‐phase encoding diffusion data were acquired, the toolbox Synb0 (Schilling, Blaber, et al., 2020) was used to generate an undistorted b0 image that served as input to FSL *eddy*.

#### 2.4.2 Generation of whole‐brain tractogram

**FIGURE 1:**
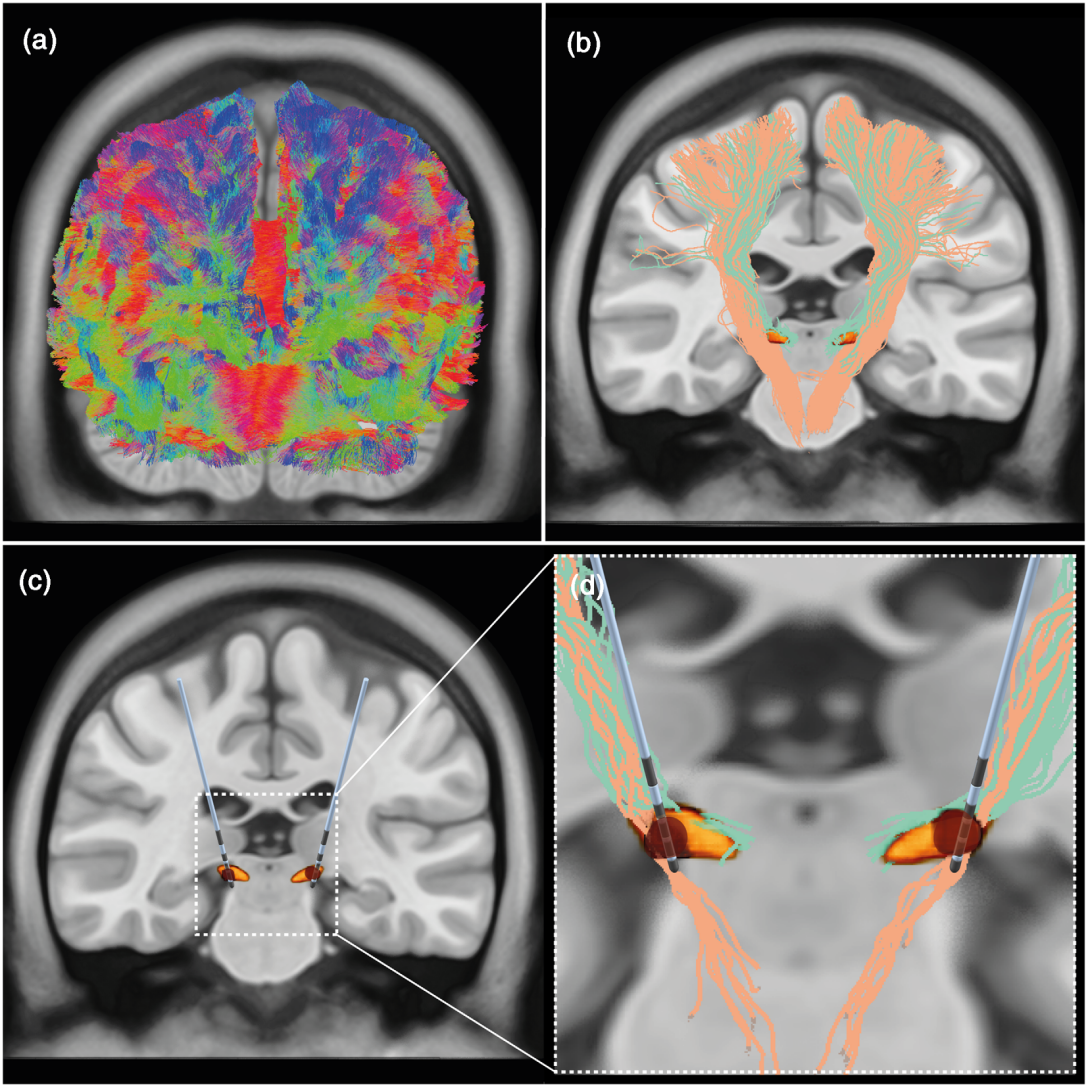
Pathway activation by DBS. (a): Generation of whole‐brain tractogram. (b): By using regions of interest and regions of avoidance, the streamlines associated with the HDP (green) and CST (salmon) were selected from the whole‐brain tractogram. STN is shown in orange. (c): Bilateral leads are localized for each patient and VTAs are generated (red volumes). (d): Streamlines from the HDP (green) and from the CST (salmon) activated by the VTAs (red). CST, corticospinal tract; DBS, deep brain stimulation; HDP, hyperdirect pathway; STN, subthalamic nucleus; VTA, volume of tissue activated.

The tractography analysis was implemented in MRtrix3 (Jeurissen et al., 2014) in the patient‐specific dMRI space. After estimating the group‐average intensity normalization (Raffelt et al., 2012) on the pre‐processed diffusion images, voxel‐wise estimates of fiber orientation distribution functions were generated with constrained spherical deconvolution (Tournier et al., 2007). For each patient, a whole‐brain tractogram of 10 million streamlines was created with the probabilistic streamline algorithm iFOD2 (Figure 1a) (Tournier et al., 2010). The anatomically constrained tractography framework (Smith et al., 2012) was included to improve the tractography results by incorporating anatomical information. Dynamic seeding (Smith et al., 2015) was used to place seed points dynamically using the SIFT model. Finally, the “spherical‐deconvolution informed filtering of tractograms (SIFT2)” method was applied to provide the streamlines' reconstruction with quantitative properties (Smith et al., 2015). By taking a whole‐brain tractogram as an input, the SIFT2 algorithm modifies the streamlines' reconstruction so that the local streamlines' densities become consistent with the densities of underlying fibers seen in the diffusion images (Smith et al., 2022). As a result, the algorithm ascribes a weight to each streamline of the whole‐brain tractogram.

#### 2.4.3 Tractography of the HDP

The HDP streamlines were extracted from the whole‐brain tractogram (Figure 1b) using Brodmann areas (BA) 4 (i.e., primary motor cortex) and 6 (i.e., supplementary motor area) (Freesurfer Brodmann area parcellation) and the STN (DISTAL atlas; Ewert et al., 2018) as regions of interest. The internal and external globus pallidus and the red nucleus (DISTAL atlas; Ewert et al., 2018), the substantia nigra (Human Motor Thalamus Atlas; Ilinsky et al., 2018), and the striatum (Oxford‐GSK‐Imanova Structural Striatal Atlas; Tziortzi et al., 2014) were used as regions of avoidance. To accept streamlines as HDP, these had to start in BAs 4 and 6 and terminate in the STN. A maximum length of 90 mm was used to avoid tracts passing through the STN and ending in other subcortical regions. All regions of interest and regions of avoidance were warped from the MNI space, where the atlases were defined, to the patient‐specific dMRI space.

#### 2.4.4 Tractography of the corticospinal tract

For obtaining the CST (Figure 1b), the following structures were used as regions of interest: BAs 4 and 6; superior corona radiata, posterior limb of internal capsule, cerebral peduncle and corticospinal tract region (JHU DTI‐based white‐matter atlas; http://www.bmap.ucla.edu/portfolio/atlases/ICBM_DTI-81_Atlas/); and medulla (Iglesias et al., 2015). The medial lemniscus and corpus callosum regions (JHU DTI‐based white‐matter atlas) and the cerebellum (Freesurfer Desikan parcellation) were used as regions of avoidance. Additionally, a minimum length of 80 mm and a maximum length of 135 mm were used.

### 2.5 Pathway‐guided programming

#### 2.5.1 Stimulation models

**FIGURE 2:**
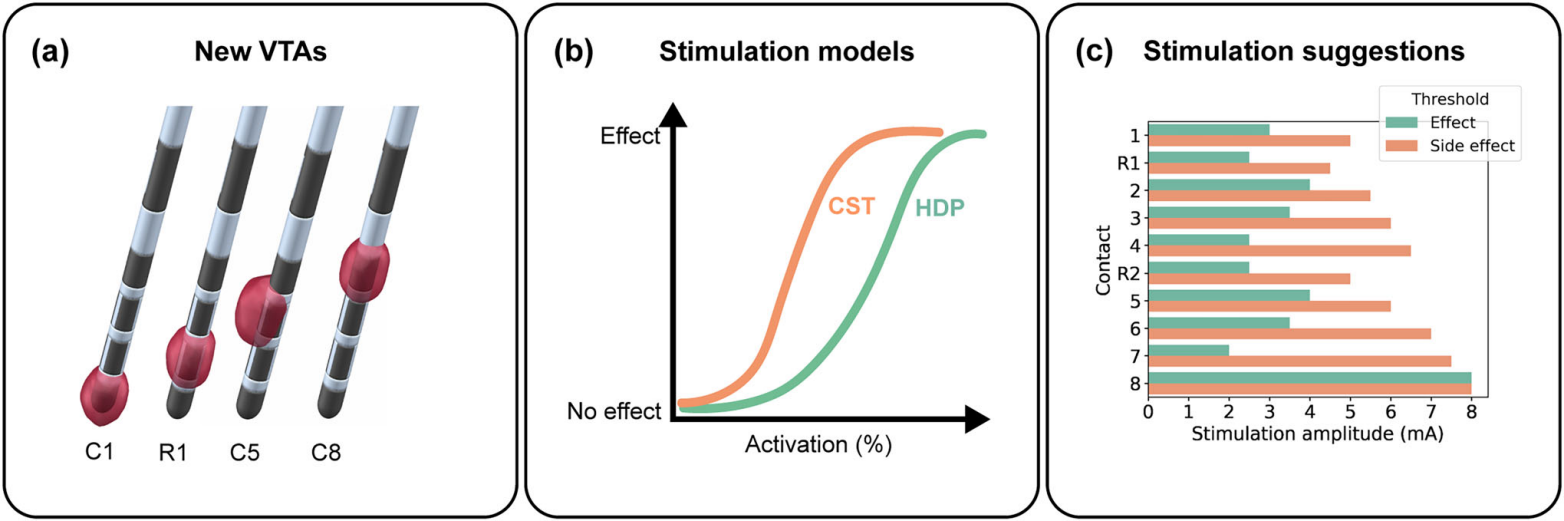
DBS parameter suggestion. (a): Generation of VTAs for all contacts in a lead. (b): Stimulation models generated with the VTAs from the monopolar review. (c): By feeding VTAs into the stimulation models, we obtain effect and side effect threshold suggestions for all the contacts in a lead. R1 and R2 correspond to the stimulation in pseudoring mode at the second and third level of the lead (i.e., R1 for contacts 2–3–4 and R2 for contacts 3–4–5). DBS, deep brain stimulation; VTA, volume of tissue activated.

Two stimulation models were calculated, one for the HDP and another for the CST using binary logistic regression. The independent variable was the activation of HDP or CST and the binary dependent variable was “no effect” or “effect” (Figure 2b). To estimate the activation of the pathways, we first identified the HDP and CST streamlines inside a given VTA (similar to other DBS structural connectivity studies, e.g., Horn et al., 2017; Li et al., 2020) and calculated the sum of the weights of those streamlines. Then we obtained the activation percentage by dividing the sum of the weights of the streamlines inside the given VTA by the total sum of the weights of the full HDP and CST, respectively.

For “no effect,” we used VTAs at 0.5 mA for each contact. We chose this value to have some minimal activation without clinical effect and to add some noise and make the model training more robust. In contrast, choosing 0 mA for “no effect” would create samples that would all fall onto 0 percent activation.

For “effect,” we used the VTAs corresponding to the stimulation thresholds for full effect from the monopolar reviews. For each contact, we used the VTA at effect threshold to determine the activation of the HDP and at side effect threshold to determine the activation of the CST.

#### 2.5.2 Cross‐validation and parameter suggestions

We evaluated the two stimulation models with nested cross‐validation (leave‐one‐subject‐out) to select the best hyperparameters. We obtained the models' outcome prediction (i.e., “effect” or “no effect”) for all VTAs of the left‐out subjects. Finally, we used the models' outcome predictions to suggest DBS parameters for the best lead level and contact, worst level and contact, effect threshold and side effect threshold (Figure 2c).

We started with an initial suggestion using the stimulation models of the HDP and the CST separately. First, we suggested the best level according to the HDP model. To obtain the best level, we combined the directional contacts of a level to one pseudoring. Second, we suggested the best contact and considered the directional contacts as individual contacts. We labeled the level and contact with the lowest effect threshold as best level and contact of that lead. Then, we compared the best level and contact suggestion from the model to the best level and contact from the monopolar review (also defined by the lowest effect thresholds). Similarly, we suggested the worst level and worst contact with the CST model. In this case, the level and the contact with the lowest stimulation thresholds resulting in capsular side effects were defined as worst level and worst contact, respectively.

In a second step, we performed a combined suggestion using both stimulation models together. First, we only kept those suggestions for which the suggested therapeutic effect threshold was at most 1 mA larger than the suggested side effect threshold. By doing this, the model would not suggest levels or contacts that had side effects before full therapeutic effect. From those remaining levels and contacts, we suggested the best level and best contact using the lowest effect threshold.

In a third step, we suggested the best level and best contact using the therapeutic window (side effect thresholds minus effect threshold) as selection criterion.

To test whether the model suggestions were significantly better than random suggestions, we performed a permutation test. We permuted the vector containing the model's suggestions and calculated the balanced accuracy for 100,000 permutations. Then we applied a right‐tailed test and calculated the *p*‐value of the model suggestions. We used the balanced accuracy as the test statistic due to our highly unbalanced classes (i.e., one best level and one best contact per lead but three non‐best levels or seven non‐best contacts per lead). This classification metric is the arithmetic mean of the sensitivity (i.e., true positive rate) and the specificity (i.e., true negative rate) and is appropriated when one of the target classes appears a lot more than the other, as it is in our case. We calculated the balanced accuracy as expressed by Equation (1) (TP: true positives, TN: true negatives, FP: false positives, FN: false negatives):

(1) $$\text{Balanced acccuracy}=0.5\times \left(\frac{\mathrm{TP}}{\mathrm{TP}+\mathrm{FN}\;}+\frac{\mathrm{TN}}{\mathrm{TN}+\mathrm{FP}}\right)$$

Finally, we compared the stimulation thresholds of the monopolar review with the models' threshold suggestion (i.e., lowest stimulation amplitude resulting in therapeutic effect or side effect). We obtained the threshold suggestion error as absolute difference between model threshold and monopolar review threshold.

### 2.6 Comparison with normative tract atlases

To compare our approach (i.e., tractography of the HDP and CST, VTA modeling, and logistic regression classifiers), we obtained the stimulation models and DBS parameter suggestions using normative tract atlases. Using the HDP from the Middlebrooks et al. (2020) tract atlas and the CST from the Meola et al. (2016) tract atlas with Lead‐DBS, we determined the streamline counts and percentages of the HDP and CST passing through the VTAs. Similar to the stimulation models using patient‐specific tractography, we related the percentage of activation to the clinical observations with logistic regression models. Finally, we performed the parameter suggestions. All these computations were done in MNI template space and not warped into patient space.

### 2.7 Data analysis

Data analysis was performed in Python. For the stimulation models, we used the *scikit‐learn* library (version 1.0.2). We used *GridSearchCV* for hyperparameter selection and to train the models with cross‐validation. Permutation tests against random selection were performed with in‐house built code.

## 3 RESULTS

### 3.1 Patient‐specific tractography

For all 20 patients, we reconstructed the whole‐brain tractogram and obtained the streamlines corresponding to the HDP and CST in both hemispheres. The streamlines corresponding to the HDP started in the motor cortex (BAs 4 and 6) and generally ended in the dorsolateral part of the STN (Supporting Information Figures S1 and S2). The streamlines from the CST also started in BAs 4 and 6 but descended ipsilaterally through the posterior limb of internal capsule and cerebral peduncle to the brainstem.

### 3.2 Stimulation models

**FIGURE 3:**
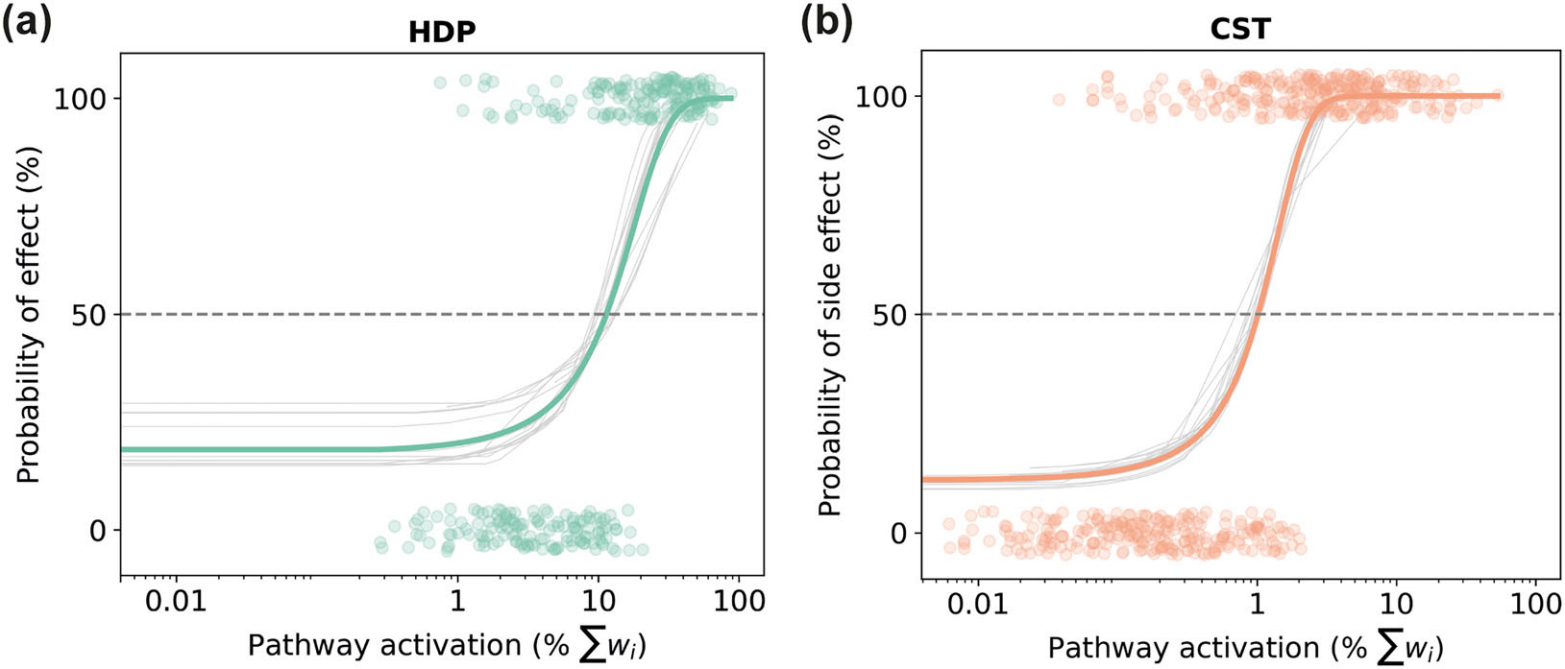
Stimulation models with weighted streamlines. (a): Hyperdirect pathway (HDP) model for therapeutic effect. (b): Corticospinal tract (CST) model for capsular side effects. Logistic regression curves differentiate between ‘no effect’ (0% probability of effect) and ‘effect’ (100% probability of effect). Gray curves correspond to the individual fits in the leave‐one‐subject‐out cross‐validation, and colored curves represent the average fit for all subjects.

DBS leads were localized for all patients (Supporting Information Figure S3) and VTAs were computed to calculate the pathway activation percentages. Then, stimulation models for the HDP and CST were obtained with logistic regression to classify the VTAs as “no effect” (0% probability of effect) or “effect” (100% probability of effect) (Figure 3). The models' classification accuracies for the train and test sets are shown in Table 1. The models' parameters (i.e., coefficients, intercept, and odds ratio) are listed in Supporting Information Table S3.

**TABLE 1:** Average classification accuracy and 95% confidence interval (CI) of the HDP and CST logistic regression models with patient‐specific tractography.

	Train set		Test set	
	Average accuracy (%)	95% CI	Average accuracy (%)	95% CI
HDP model	83.2	82.9–83.5	79.4	72.7–86.2
CST model	84.6	84.3–84.8	84.2	78.2–90.2

Abbreviations: CST, corticospinal tract; HDP, hyperdirect pathway.

The HDP model (Figure 3a) implies that on average 50% of the HDP must be activated for a 100% probability of effect. The CST model (Figure 3b) implies that on average, 4% of the CST must be activated for a 100% probability of capsular side effects. In both cases, the activation percentage of the pathways corresponds to the percentage of the sum of the weights.

### 3.3 Parameter suggestions

In the initial suggestions, we obtained the best and worse lead levels and contacts using the stimulation models of the HDP and CST separately. The model's suggestions for best level matched the best clinical levels with a balanced accuracy of 63.1% (*p* = .002). For the best contacts, the model's suggestions matched the best clinical contacts with a balanced accuracy of 49.6% (*p* = .602). Regarding the worst level, the model's suggestions matched the worst clinical levels with a balanced accuracy of 67.9% (*p* = 3e‐05). And for the worst contact, the model's suggestions matched the worst clinical contacts with a balanced accuracy of 65.1% (*p* = .0001).

In the combined suggestions, we suggested the best levels and contacts using both stimulation models together. We excluded “best” level or contact suggestions where capsular side effects would appear before therapeutic effect. The suggestions for best level matched the best clinical levels with an accuracy of 62.2% (*p* = .003). For the best contacts, the model's suggestions matched the best clinical contacts with an accuracy of 47.7% (*p* = .771).

When using the therapeutic window as selection criterion, the suggestions for best level matched the best clinical levels with an accuracy of 63.9% (*p* = .001). For the best contacts, the model's suggestions matched the best clinical contacts with an accuracy of 51.4% (*p* = .407). All values for sensitivity (i.e., true positive rate), specificity (i.e., true negative rate), balanced accuracy and *p*‐values of the permutation tests are listed in Table 2.

**TABLE 2:** Classification metrics for the models' suggestions with patient‐specific tractography.

			Sensitivity (TPR)	Specificity (TNR)	Balanced accuracy (%)	*p*‐value of permutation test
Initial suggestions	HDP	Best level	0.58	0.68	63.1	**.002**
		Best contact	0.36	0.63	49.6	.602
	CST	Worst level	0.69	0.67	67.9	**3e‐05**
		Worst contact	0.62	0.68	65.1	**.0001**
Combined suggestions	HDP and CST	Best level	0.54	0.71	62.2	**.003**
		Best contact	0.32	0.64	47.7	.771
Therapeutic window	HDP and CST	Best level	0.51	0.76	63.9	**.001**
		Best contact	0.24	0.79	51.4	.407

*Note*: Balanced accuracy scores account for the percentage of suggested best and worst levels and contacts that match the clinical classification. Bold *p*‐values correspond to significant results.

Abbreviations: CST, corticospinal tract; HDP, hyperdirect pathway; TNR: true negative rate; TPR: true positive rate.

As the best contact suggestions were statistically non‐significant, we calculated the distance between the best contact suggestions of the model and the best contact of the monopolar review. For the best contact suggestions, half of the wrongly suggested contacts were at the same level as the best clinical contacts (Supporting Information Tables S8 and S10).

**FIGURE 4:**
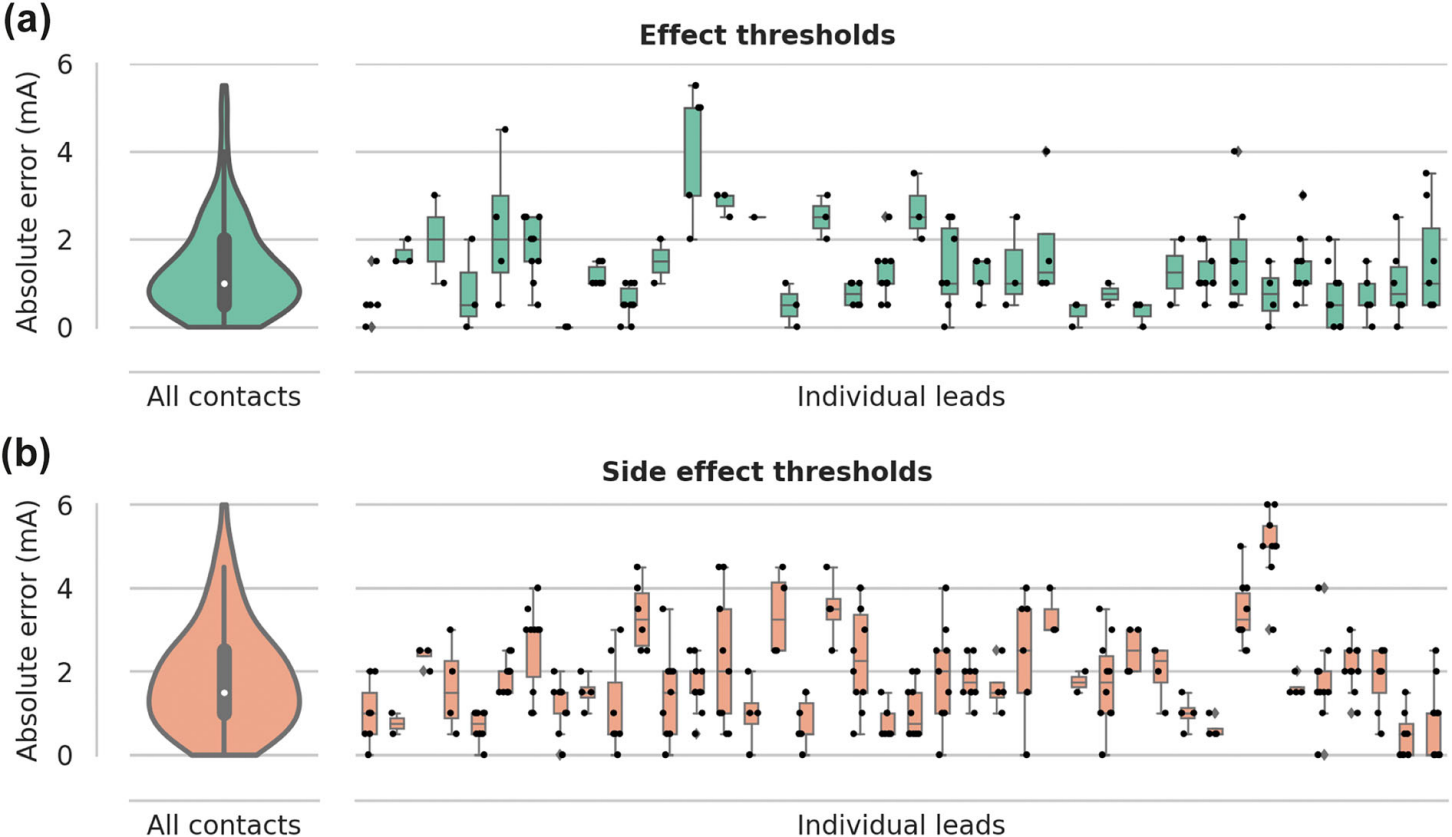
Absolute threshold suggestion error in leave‐one‐subject‐out cross‐validation. (a): Threshold suggestion error for effect (activation of HDP). (b): Threshold suggestion error for side effect (activation of the CST). Left panels contain violin plots with the absolute threshold errors for all contacts; miniature boxplots show the quartiles and the median value. Right panels contain boxplots for individual leads with scatter points showing individual contact errors. CST, corticospinal tract; HDP, hyperdirect pathway.

Finally, we compared the suggested effect thresholds and side effect thresholds to the thresholds from the monopolar reviews. The absolute errors for all leads are shown in Figure 4. The median error for all VTAs was 1 mA for the effect thresholds and 1.5 mA for the side effect thresholds. We also analyzed the signed error and did not find systematic overestimation or underestimation by the models (Supporting Information Figure S5).

### 3.4 Stimulation models with normative tract atlases

We obtained the stimulation models and the parameter suggestions with the normative tract atlases. The activation threshold at 50% probability of effect was considerably larger than with patient‐specific tractography (i.e., 30% for the HDP and 35% of CST versus 10% and 0.9%, respectively). In both models, for a 100% probability of effect, all the streamlines in the pathway had to be activated (Supporting Information Figure S6). The models classification accuracies for the train and test sets are shown in Table 3. The models' parameters (i.e., coefficients, intercept and odds ratio) are listed in Supporting Information Table S4.

The atlas‐guided suggestions performed similarly well as the model suggestions based on patient‐specific tractography (e.g., balanced accuracy of 62.9% for best level; all values listed in Table 4). However, using the normative tract atlases was more accurate to suggest the best contact for therapeutic window (60.1% vs. 51.4%).

**TABLE 3:** Average classification accuracy and 95% confidence interval (CI) of the HDP and CST logistic regression models with normative tract atlases.

	Train set		Test set	
	Average accuracy (%)	95% CI	Average accuracy (%)	95% CI
HDP model	78.4	78.0–78.8	72.4	65.3–78.8
CST model	87.1	86.8–87.5	48.6	47.0–50.2

Abbreviations: CST, corticospinal tract; HDP, hyperdirect pathway.

Finally, we compared the suggested effect thresholds and side effect thresholds to the thresholds from the monopolar reviews. The absolute errors for all leads are shown in Supporting Information Figure S8. The median error for all VTAs was 1 mA for the effect thresholds and 3 mA for the side effect thresholds. The signed errors did not show a systematic overestimation or underestimation by the models (Supporting Information Figure S9).

## 4 DISCUSSION

This study used patient‐specific tractography and stimulation models of the HDP and CST to suggest DBS parameters. The models suggested the best levels more accurately than random suggestions. Suggestions of the best contacts and the effect and side effect thresholds proved to be more challenging and warrants further investigations.

### 4.1 Patient‐specific tractography

Here, we performed patient‐specific tractography to obtain the HDP and CST. Other studies substitute patient dMRI data with tractography atlases (Petersen et al., 2019) or with normative connectome data, where brain connectivity is calculated and averaged from a large cohort of subjects (Wang et al., 2021). The use of normative connectomes or tractography atlases is motivated by the lack of or the lower quality of clinical routine dMRI data and data heterogeneity across centers. But the main limitation of atlases or normative connectomes is that they may not be able to describe individual differences in connectivity profiles.

One limitation of probabilistic tractography on patient‐specific dMRI is the high number of false‐positive connections (Petersen et al., 2019; Schiavi et al., 2020; Schilling, Petit, et al., 2020). It has been shown that manually placed or template‐driven constraints can improve the anatomical accuracy of the estimated connections (Schilling, Petit, et al., 2020). Therefore, we defined regions of interest and regions of avoidance to improve the accuracy of the HDP and CST tractography. Both our pathways were in general accordance with the literature of anatomical tracing and tractography (Emmi et al., 2020; Temiz et al., 2020; Welniarz et al., 2017). In addition, to improve the accuracy and the quantitative properties of the tractography results, we used an advanced technique with constrained‐spherical deconvolution (Jeurissen et al., 2014). We estimated the fiber orientation distribution per voxel, and performed anatomically constrained tractography (Smith et al., 2012), dynamic seeding and filtering with SIFT2 (Smith et al., 2015). The use of filtering algorithms such as SIFT2, which assigns weights to the streamlines, addresses the limitations of streamlines counts and enables quantitative assessment of fiber connectivity (Smith et al., 2022).

### 4.2 Stimulation models with patient‐specific tractography

#### 4.2.1 HDP stimulation model

In the HDP model, an activation of 50% of the sum of the weights resulted in a 100% probability of therapeutic effect. This suggests that the HDP is highly activated at maximal rigidity reduction. A previous study reported an activation for the HDP of 13.6 ± 1.2% of the *streamline counts* in one patient at the clinical effective stimulation setting (Gunalan et al., 2017). However, the activation of the HDP was constrained by stimulation spread into the internal capsule fibers of passage (Gunalan et al., 2017). In a more recent study, Noecker et al. (2021) developed a DBS activation model containing nine general pathways from a pathway atlas (Petersen et al., 2019). They analysed three STN DBS patients and observed that the directional contacts located at the dorsal border of the STN activated a wider range of pathways, including the internal capsule fibers of passage. Their results showed a large variance in the inter‐patient activation percentages and no consistent activation of the motor HDP (Noecker et al., 2021).

Based on 20 patients, our activation of the motor HDP was considerably larger and more consistent than reported by Gunalan et al. (2017) and Noecker et al. (2021). This value corresponded to the percentage of the sum of the weights of HDP required for a 100% probability of therapeutic effect. For a 50% probability of therapeutic effect, our model suggested an activation threshold slightly above 10%, which would be in line with the 13.6% of activation obtained by Gunalan et al. (2017). Contrary to the results from Noecker et al. (2021), we observed a consistent activation of the motor HDP: 76.4% of the VTAs at maximal therapeutic effect had an activation of the HDP above 10%.

Differences in the activation thresholds can be due to many factors. The streamline generation (patient‐specific tractography vs. normative tract atlas, the selection of included pathways) and differences in pathway activation modeling (streamlines activated by the VTA versus axon cable models) may be two main factors.

#### 4.2.2 CST stimulation model

In the CST model, an activation of 4% of the sum of the weights resulted in a 100% probability of capsular side effects. For a 50% probability, the activation threshold was at 1%. This suggests that even small percentages of CST activation would lead to capsular side effects. The models of Gunalan et al. (2017) and Noecker et al. (2021) reported the activation of CST fibers at clinical DBS settings. In Gunalan et al. (2017), there was no activation of the internal capsule fibers of passage at the effective stimulation setting; in Noecker et al. (2021), only the best responder patient had a stimulation of the motor internal capsule fibers less than 2%. To our knowledge, there are no other studies quantifying the activation of the CST and relating it to the side effect thresholds by means of tractography and stimulation models.

### 4.3 Parameter suggestions

The stimulation models predicted the best stimulation levels with an accuracy above 60%, and the best stimulation contacts with an accuracy of around 50% (Table 2). To test the statistical significance of the models' suggestions, we performed permutation tests (Supporting Information Figures S4 and S7). In the permutation tests, we kept the same number of best levels and best contacts as in the models' original suggestions. Given the unbalanced data set (very few best levels, contacts), the permutation tests were more stringent than a binomial test. With a binomial test assuming one best contact per lead, our initial best contact suggestions would have been statistically significant (*p* < .05).

**TABLE 4:** Classification metrics for the models' suggestions with normative tract atlases.

			Sensitivity (TPR)	Specificity (TNR)	Balanced accuracy (%)	*p*‐value of permutation test
Initial suggestions	HDP	Best level	0.60	0.66	62.9	**.002**
		Best contact	0.36	0.69	52.8	.300
	CST	Worst level	0.29	0.99	64.1	**.000**
		Worst contact	0.17	0.95	56.3	**.005**
Combined suggestions	HDP and CST	Best level	0.60	0.66	62.9	**.002**
		Best contact	0.36	0.69	52.7	.308
Therapeutic window	HDP and CST	Best level	0.70	0.67	69.3	**3e‐05**
		Best contact	0.47	0.72	60.1	**.009**

*Note*: Balanced accuracy scores account for the percentage of suggested best and worst levels and contacts that match the clinical classification. Bold *p*‐values correspond to significant results.

Abbreviations: CST, corticospinal tract; HDP, hyperdirect pathway; TNR, true negative rate; TPR, true positive rate.

The accuracy of best contact suggestion may have been affected by incomplete and shortened monopolar reviews. In our cohort, 60.8% of all directional contacts were tested. In particular, they were not tested when the effect threshold of the level was above the side effect threshold, or when the effect threshold of the level was less than 3 mA and there was still a margin to the side effect threshold. If all directional contacts had been tested, we would have had more data to compare with the models' suggestions, and the accuracy could have improved.

Next, we suggested effect thresholds and capsular side effect thresholds. The absolute median error for the effect threshold was 1 mA, and 1.5 mA for the side effect threshold (Figure 4). But for individual leads, we observed a higher variability. Considering average effect thresholds of 2–3 mA for STN DBS and that contacts were tested with 0.5 mA steps, these errors could result in suggestions being two or three steps away from the optimal threshold. These differences could be due to errors in the lead reconstruction, in the patient‐specific tractography, and due to the VTA generation model.

We also computed parameter suggestions with normative tract atlases and compared them to our suggestions with patient‐specific tractography. We expected normative tract atlases to have lower variability than tracts derived from patient‐specific tractography. This might have led to more accurate suggestions. But that was not the case and the suggestions for best levels had similar balanced accuracies. On the other hand, the suggested side effect thresholds were less accurate with the normative tract atlases than for the patient‐specific tractography models. In the normative tract atlas model, an activation of 35% of the pathway was required to classify a VTA as side effect. As a result, the model only found capsular side effects in 58 out of the 276 settings. In the HDP models, the difference between patient‐specific tractography and normative tract atlases was less clear. This can be due to the reliability of the patient‐specific HDP tractography or the assumption of the HDP as sole mediator of rigidity improvement. Based on our findings, we believe that patient‐specific tractography, with all its limitations, can be a valuable tool in DBS programming, in particular to reduce the probability of side effects.

Other approaches to facilitate DBS programming include, for instance, probabilistic stimulation maps (Dembek et al., 2019; Nguyen et al., 2019), image‐based automated algorithms (Roediger et al., 2021), and electrophysiology (Miocinovic et al., 2018; Shah et al., 2022; Tinkhauser et al., 2018). Probabilistic stimulation maps have demonstrated a correlation between sweet spot activation and clinical improvement. Using the overlap between a VTA and the sweet spot, these models try to predict the clinical outcome of a given VTA. Contrary to our study, the prediction models based on sweet spots rely on the voxel‐based statistical analysis of a cohort of patients rather than incorporating patient‐specific tractography. The StimFit algorithm also suggests stimulation settings (Roediger et al., 2021). This method also had a moderate degree of correlation between the suggestions and the empirical clinical settings. Finally, electrophysiological recordings have also been used to identify the most effective stimulation contacts, with an accuracy similar to our models (Shah et al., 2022; Tinkhauser et al., 2018).

However, suggestions may not need to perfectly match clinical settings and yet have similar clinical improvement. Therefore, prospective studies are needed to evaluate the motor improvement of a model's suggestions.

### 4.4 Limitations and future work

Our study has several limitations. One major limitation of using patient‐specific dMRI is data quality. Fiber tractography bundle segmentation will depend on the scanner acquisition and the processing pipeline (Schilling et al., 2021). Care must be taken when deriving and interpreting quantitative measures of connectivity. Besides, the stimulation models should be further validated with patient‐specific tractography obtained from other dMRI datasets to test its reliability.

Our pathway stimulation models relied on VTAs to estimate the stimulation volume and recruited streamlines. But VTA models might have limitations in the subthalamic region (Noecker et al., 2021). In our models, we did not consider different activation thresholds for the two pathways (i.e., HDP and CST). But recent work from Bower and McIntyre (2020) showed that HDP terminating axons have a lower activation threshold than fibers of passage and not accounting for these differences may be influencing our models' activation thresholds. VTA generation models implementing driving‐force methods could better estimate the response of complex axonal pathways to DBS (Noecker et al., 2021).

The definition of the HDP is not fully established yet (Bingham et al., 2023). It has generally been described as collateral fibers of corticofugal axons descending to lower brainstem regions. In our study, we reconstructed this pathway as streamlines starting in the motor cortex and ending in the STN. Other studies have included the HDP as collateral fibers of corticofugal axons, but only including collaterals with terminals in the STN (Bingham & McIntyre, 2022; Gunalan et al., 2017; Petersen et al., 2019). However, single‐axon tracing studies in the rat (Kita & Kita, 2012) and in primates (Coudé et al., 2018) observed that around half of the collaterals innervating the STN continued to the zona incerta and other brain regions. The reconstruction of the HDP with patient‐specific tractography, as performed in our study and by Petersen et al. (2017), is limited by the tractography approach itself (i.e., by definition, a streamline has a single start point and end point) and collaterals from an individual streamline cannot be obtained. Since the available histological data are suggesting that collaterals with terminals in the STN are only a fraction of the HDP, future models should also investigate the HDP's more intricate anatomy (Bingham & McIntyre, 2022) and terminals in regions beyond the STN.

Although previous studies suggested an important role of the HDP for therapeutic effect (Miocinovic et al., 2018; Oswal et al., 2021), the activation of other white matter tracts and connectivity to other basal ganglia nuclei might also be influential. Neither previous studies nor our study modeled the activation of the indirect pathway. This pathway may also mediate the therapeutic effect. It connects the STN to the globus pallidus and it runs in a similar anatomical space as the HDP (Weaver et al., 2020). But the methods used in the present study cannot sufficiently distinguish activation of the HDP from activation of the indirect pathway due to the close location of their terminals in the STN.

The accuracy of our models' suggestions for best stimulation contacts and thresholds demonstrated the challenges of tract‐based programming. While the models suggested the best levels with a balanced accuracy above 60%, which would be acceptable for DBS practice, the suggestions for contacts and thresholds had a lower accuracy and still need improvement. In the future, the stimulation models developed herein may be expanded to include more pathways to pursue personalized connectomic programming (Hollunder et al., 2022). This could then be used to provide symptom‐specific programming, that is, activation of individualized networks according to the symptom spectrum of specific patients. But prospective clinical studies are required to assess the value of these symptom‐specific suggestions.

## 5 CONCLUSION

Stimulation models of the HDP and CST were used to model therapeutic effect and side effects and suggested DBS settings with a good balanced accuracy. Improvement of dMRI and tractography as well as prospective clinical studies are warranted to optimize tract‐guided DBS programming. Together with other modalities, these may allow for assisted STN DBS programming.

## AUTHOR CONTRIBUTIONS

**Alba Segura‐Amil**: Conceptualization; methodology; software; formal analysis; investigation; writing—original draft; visualization. **Andreas Nowacki**: Conceptualization; methodology; validation; writing—review and editing. **Ines Debove**: Data curation. **Katrin Petermann**: Data curation; validation. **Gerd Tinkhauser**: Validation; writing—review and editing. **Paul Krack**: Validation; writing—review and editing. **Claudio Pollo**: Conceptualization; methodology; validation; writing—review and editing. **T. A. Khoa Nguyen**: Conceptualization; methodology; writing—review and editing; supervision; funding.

## CONFLICT OF INTEREST STATEMENT

Author Andreas Nowacki received consultant fees from Medtronic, not relevant to the submitted work. Author Ines Debove received research and travel grants from Boston Scientific, not relevant to the submitted work. Gerd Tinkhauser received financial support from Boston Scientific and Medtronic and has a research agreement with RuneLabs, all not related to the present work. Author Claudio Pollo is co‐founder of Aleva Neurotherapeutics SA and received consultant fees from Boston Scientific and Abbott, not relevant to the submitted work. The remaining authors declare that the research was conducted in the absence of any commercial or financial relationships that could be construed as a potential conflict of interest.

## Supporting information


**Data S1:** Supporting Information.

## Data Availability

Pipelines for dMRI processing and data analysis are available in: https://gitlab.switch.ch/brain‐stimulation‐mapping/2023_dbsprogramming‐hdp‐cst A de‐identified data set containing tracts in patient space, the MNI VTAs and transformations from MNI to patient space can be provided on application, subject to institutional review board approval.
