# Supplementary material for: Programming of subthalamic nucleus deep brain stimulation with hyperdirect pathway and corticospinal tract‐guided parameter suggestions
Source: Hum Brain Mapp. 2023 Jun 15;44(12):4439–51. doi: 10.1002/hbm.26390 (PMC10365233; doi:10.1002/hbm.26390)
Supplement: Supplementary file 1 — Data S1: Supporting Information. [file HBM-44-4439-s001.docx]

# Supplementary Figures


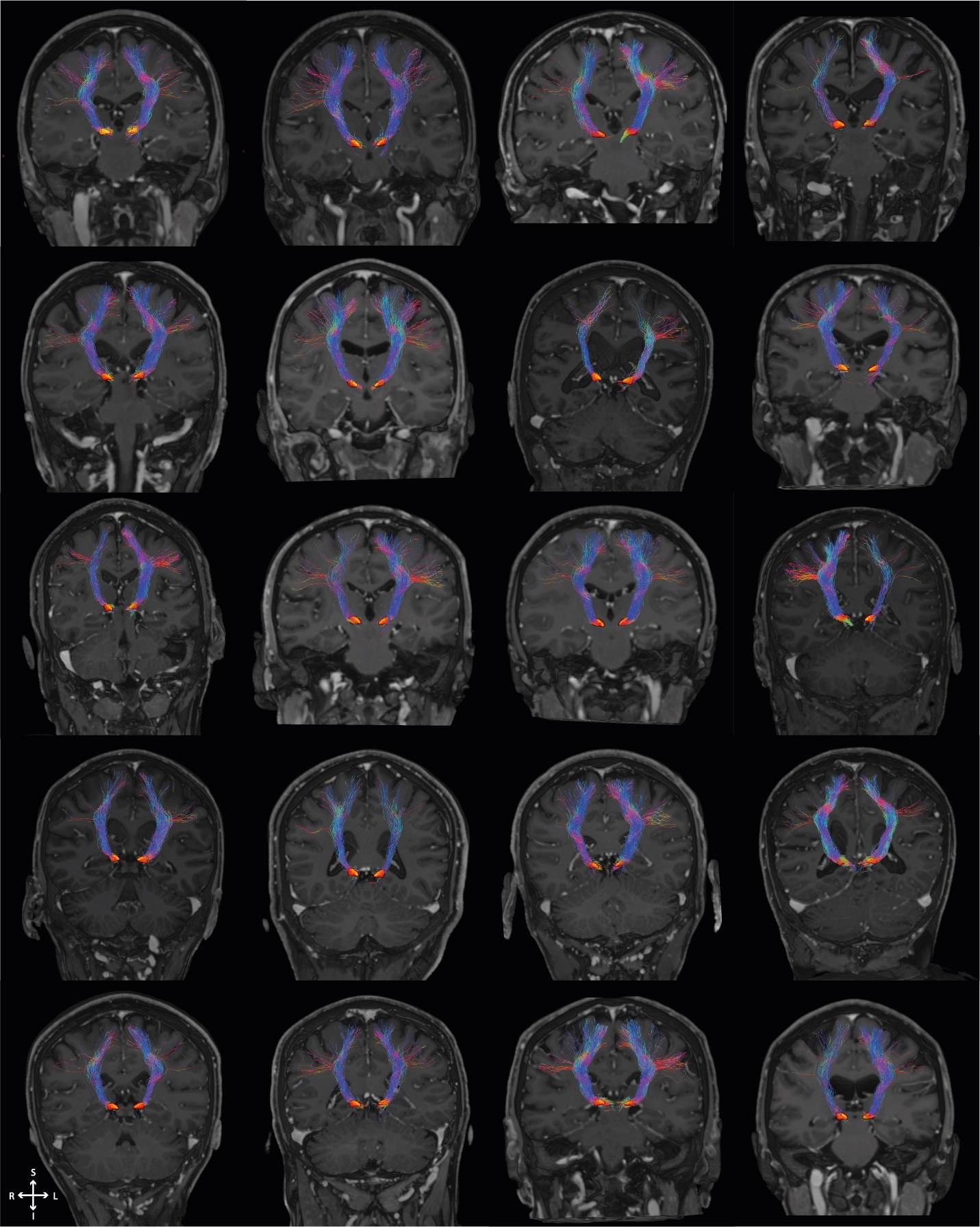


Supplementary Figure 1. Hyperdirect pathway streamlines in the subthalamic nucleus (orange) for the patient cohort (n=20).


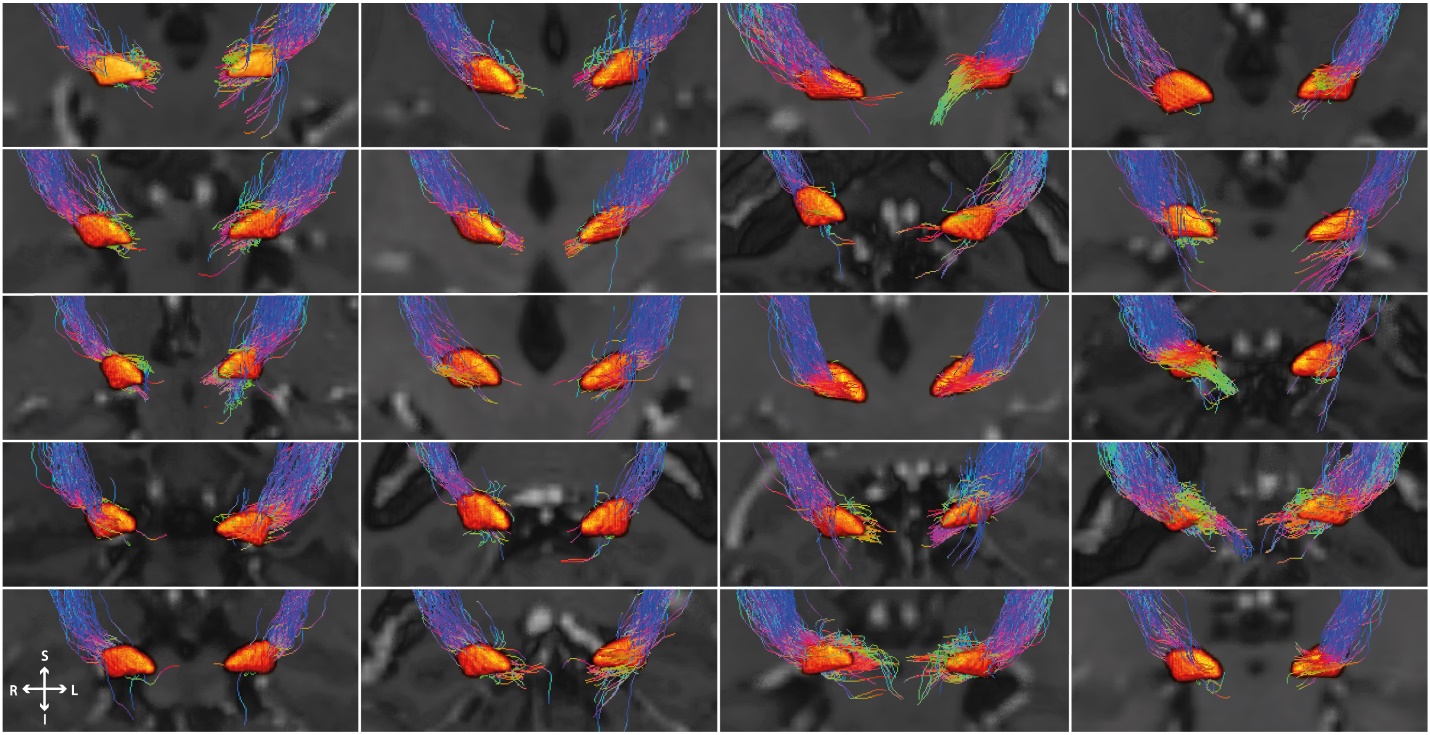


Supplementary Figure 2. Hyperdirect pathway terminals in the subthalamic nucleus (orange) for the patient cohort (n=20).


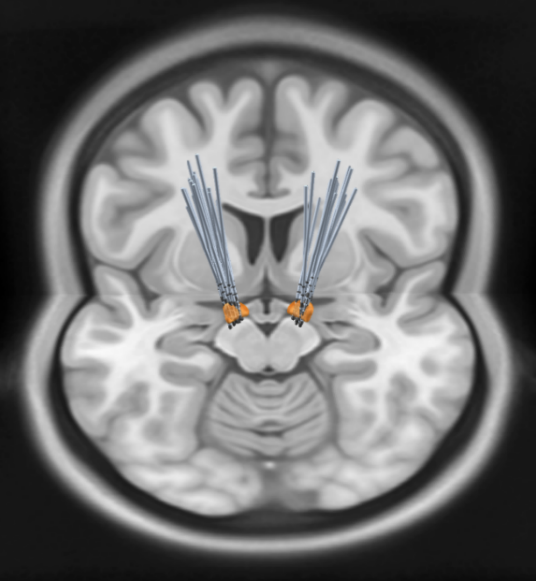


*Supplementary Figure 3. Overview of lead placement for all twenty patients in MNI space. Subthalamic nucleus is shown in orange.*

**Patient-specific tractography**


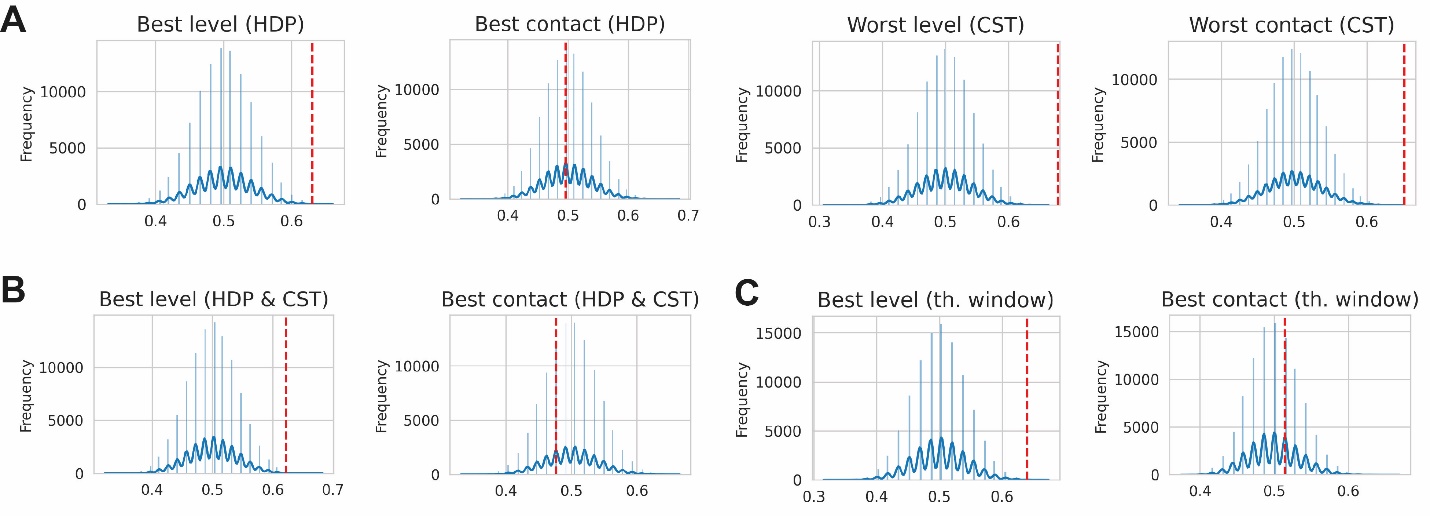


Supplementary Figure 4. Permutation test distributions with patient-specific tractography. A: initial suggestions; B: combined suggestions, C: therapeutic window suggestions. The vertical red line indicates the test statistic for the models’ suggestions.


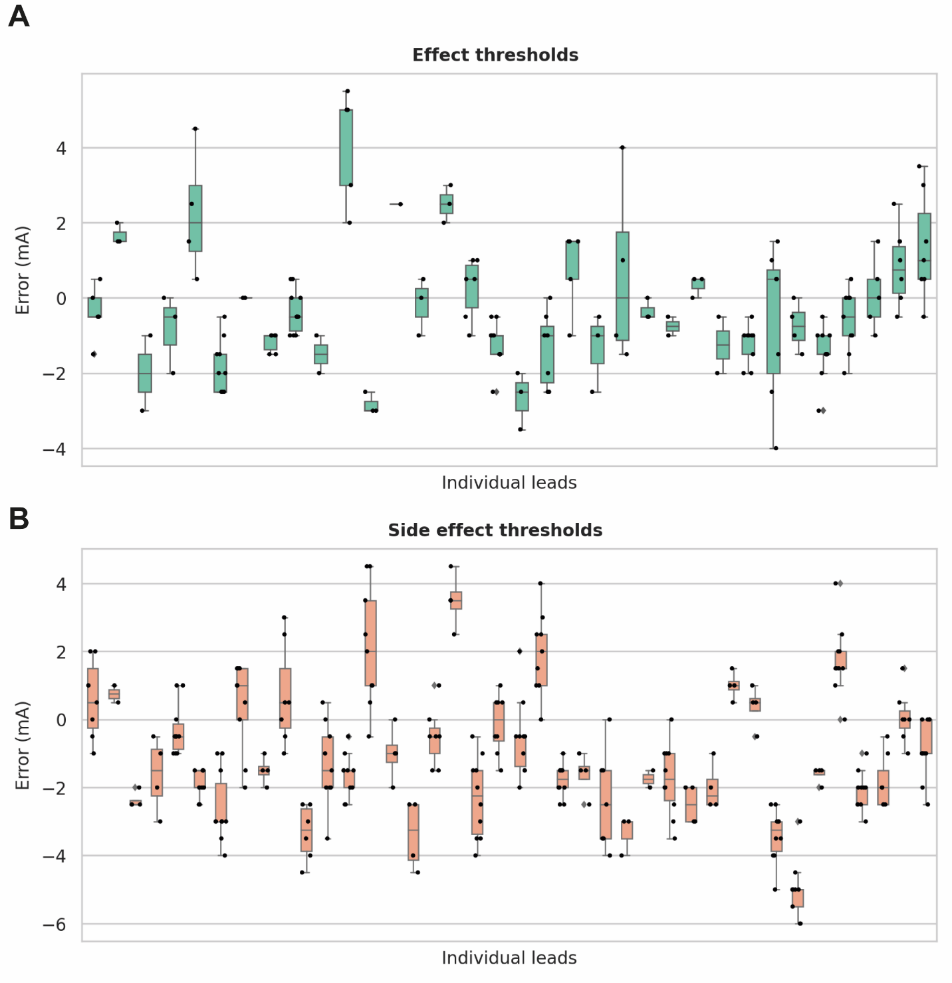


Supplementary Figure 5. Signed threshold suggestion error in leave-one-subject-out cross-validation of stimulation models with patient-specific tractography. A: Threshold suggestion error for effect (activation of HDP). B: Threshold suggestion error for side effect (activation of the CST). Boxplots for individual leads with scatter points showing individual contact errors.

**Normative tract atlases**


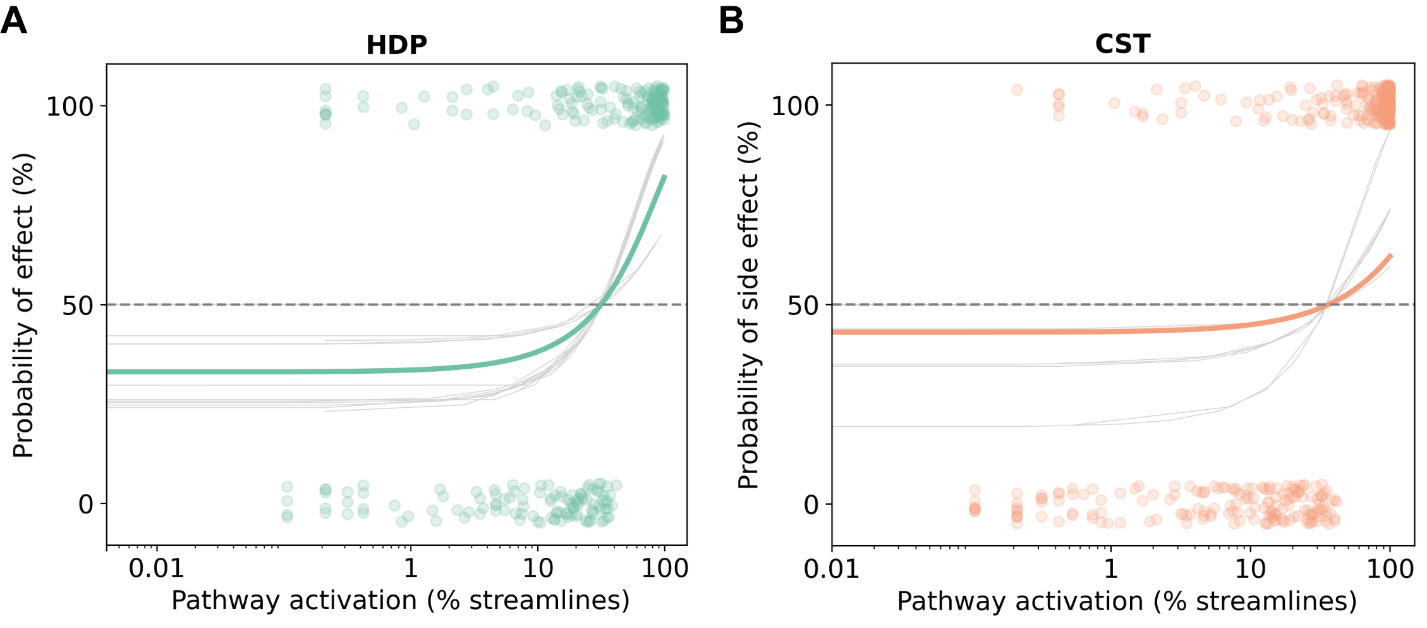


Supplementary Figure 6. Stimulation models with normative tract atlases. A: Hyperdirect pathway (HDP) model for therapeutic effect. B: Corticospinal tract (CST) model for capsular side effects. Logistic regression curves differentiate between ‘no effect’ (0% probability of effect) and ‘effect’ (100% probability of effect). Grey curves correspond to the individual fits in the leave-one-subject-out cross-validation, and colored curves represent the average fit for all subjects.


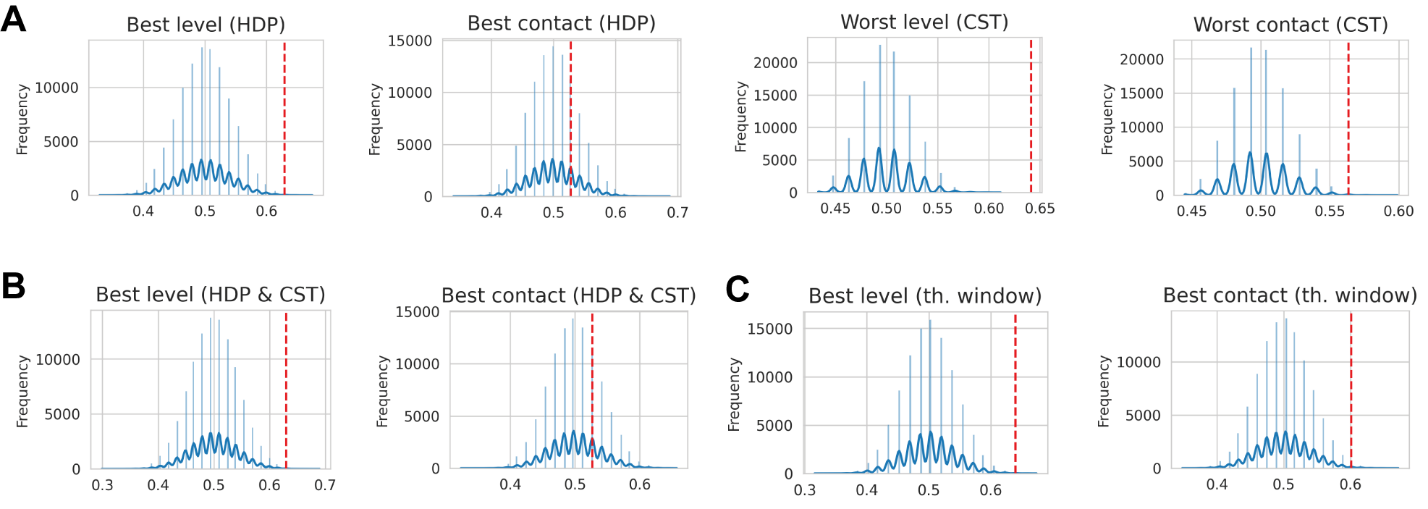


Supplementary Figure 7. Permutation test distributions with normative tract atlases. A: initial suggestions; B: combined suggestions; C: therapeutic window suggestions. The vertical red line indicates the test statistic for the models’ suggestions.


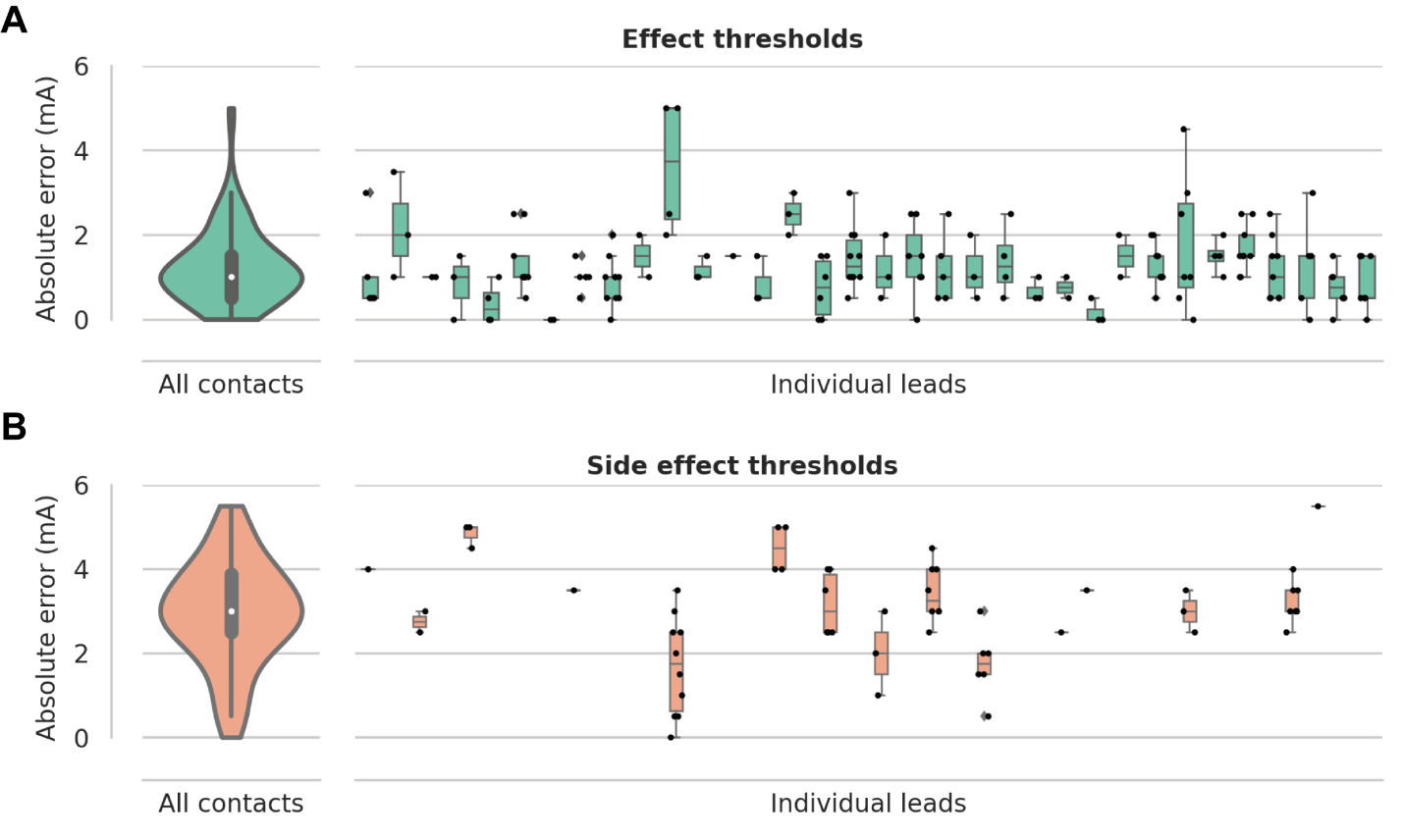


Supplementary Figure 8. Absolute threshold suggestion error in leave-one-subject-out cross-validation of stimulation models with normative tract atlases. A: Threshold suggestion error for effect (activation of HDP). B: Threshold suggestion error for side effect (activation of the CST). Left panels contain violin plots with the absolute threshold errors for all contacts; miniature boxplots show the quartiles and the median value. Right panels contain boxplots for individual leads with scatter points showing individual contact errors.


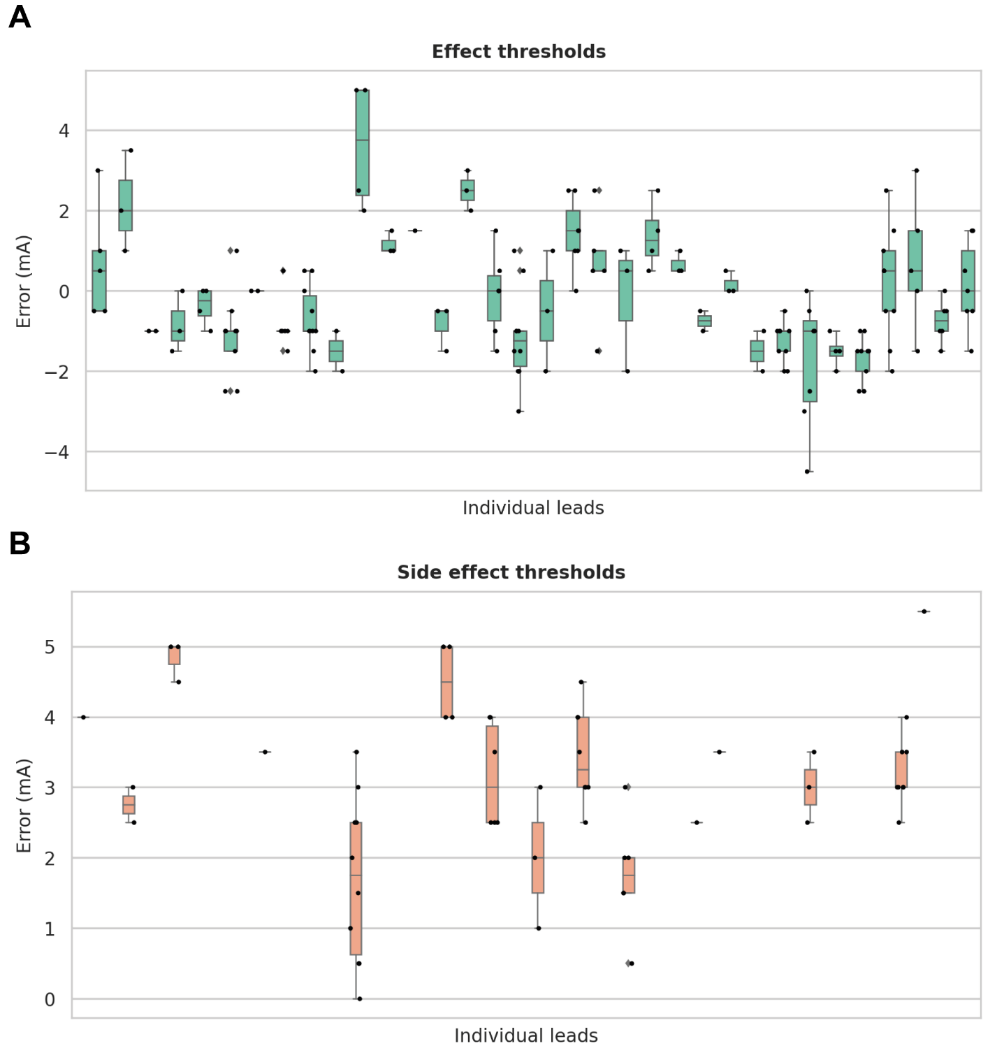


Supplementary Figure 9. Signed threshold suggestion error in leave-one-subject-out cross-validation of stimulation models with normative tract atlases. A: Threshold suggestion error for effect (activation of HDP). B: Threshold suggestion error for side effect (activation of the CST). Boxplots for individual leads with scatter points showing individual contact errors.

# Supplementary Tables

Supplementary Table 1. Number of gradient directions and corresponding b-values of the diffusion MRI acquisition.

| **n° gradient directions** | 1 | 6 | 12 | 8 | 6 | 24 | 24 | 12 | 30 |
| --- | --- | --- | --- | --- | --- | --- | --- | --- | --- |
| **B-value** | 0 | 342 | 650 | 1000 | 1333 | 1650 | 2000 | 2650 | 3000 |

*Supplementary Table 2. Stimulation data (VTAs) included for training and testing of the models.*

| **CLINICAL DATA** | |
| --- | --- |
| 20 subjects  40 leads  400 VTAs | |
| **Tested contacts:** | |
| HDP | 37 leads (3 leads with lesion effect, only VTAs at 0mA)  275 VTAs > 0mA |
| CST | 40 leads  389 VTAs capsular side effect  281 VTAs capsular side effect > 0 mA  276 VTAs capsular side effect > 0 mA & < 8mA |
| **MODEL TRAINING DATA** | |
| Include only VTAs at full effect | |
| HDP | 34 leads (3 leads with no effect, only VTAs at 8mA)  165 VTAs >0 mA & <8 mA |
| CST | 40 leads  276 VTAs >0 mA & <8 mA |
| **MODEL TESTING DATA** | |
| Use the models to make predictions on all tested leads | |
| HDP | 37 leads (3 leads with lesion effect)  5550 VTAs (150 VTAs/lead - 1...0.5...8 mA) |
| CST | 40 leads  6000 VTAs |

Supplementary Table 3. Parameters of logistic regression models with patient-specific tractography. Average values of the leave-one-subject-out cross-validation.

|  | **HDP model** | **CST model** |
| --- | --- | --- |
| **Coefficient** | 0.13 | 1.95 |
| **Intercept** | -1.50 | -1.98 |
| **Odds Ratio** | 1.14 | 7.04 |

Supplementary Table 4. Parameters of logistic regression models with normative tract atlases. Average values of the leave-one-subject-out cross-validation.

|  | **HDP model** | **CST model** |
| --- | --- | --- |
| **Coefficient** | 0.02 | 0.01 |
| **Intercept** | -0.71 | -0.28 |
| **Odds Ratio** | 1.02 | 1.01 |

*Supplementary Table 4. Sum of weights of the hyperdirect pathway (HDP) and corticospinal tract (CST). HDP and CST were reconstructed in each patient individually, and weights values were obtained after applying the SIFT2 algorithm.*

| **Lead ID** | **Sum weights HDP** | **Sum weights CST** |
| --- | --- | --- |
| 1 | 283.5 | 1469.6 |
| 2 | 154.8 | 615.4 |
| 3 | 186.3 | 5105.4 |
| 4 | 173.7 | 3602.0 |
| 5 | 491.8 | 9557.5 |
| 6 | 134.8 | 2455.9 |
| 7 | 167.0 | 9241.2 |
| 8 | 54.4 | 8211.3 |
| 9 | 245.9 | 5718.5 |
| 10 | 168.0 | 3978.3 |
| 11 | 267.5 | 10356.2 |
| 12 | 184.6 | 7503.3 |
| 13 | 93.5 | 2505.2 |
| 14 | 42.9 | 1093.6 |
| 15 | 96.3 | 7272.3 |
| 16 | 234.3 | 8737.3 |
| 17 | 383.6 | 3962.8 |
| 18 | 68.1 | 2364.9 |
| 19 | 233.7 | 4191.8 |
| 20 | 61.3 | 4318.2 |
| 21 | 236.0 | 3477.0 |
| 22 | 136.3 | 2250.1 |
| 23 | 39.2 | 4841.9 |
| 24 | 538.3 | 1457.4 |
| 25 | 160.4 | 3735.0 |
| 26 | 100.5 | 2652.4 |
| 27 | 46.6 | 9541.0 |
| 28 | 89.1 | 9135.3 |
| 29 | 324.0 | 4508.1 |
| 30 | 151.1 | 4909.6 |
| 31 | 241.1 | 4751.6 |
| 32 | 371.4 | 15317.7 |
| 33 | 110.7 | 939.2 |
| 34 | 92.0 | 800.0 |
| 35 | 125.9 | 7808.6 |
| 36 | 80.2 | 4582.4 |
| 37 | 245.0 | 5152.1 |
| 38 | 266.1 | 3351.5 |
| 39 | 218.8 | 3181.6 |
| 40 | 48.7 | 4922.6 |

Description for Supplementary Tables 5-11:

- All matching: all levels/contacts in the monopolar review were correctly suggested by the model
- Matching & extra suggestions: best/worst levels/contacts in the monopolar review were suggested by the model; additional levels/contacts were also suggested as best/worst
- Matching & missing suggestions: at least one best/worst level/contact in the monopolar review was suggested by the model; some levels/contacts were not suggested as best/worst
- None matching: none of the best/worst levels/contacts were suggested by the model
- No best clinical levels: Leads without best levels/contacts in the monopolar review
- No best model levels: Leads without best levels/contacts in model’s suggestions
- Total leads: number of leads considered (see Supplementary table 1)

Supplementary Table 5. Level deviations in HDP model’s suggestions compared to best levels in the clinical monopolar review. Numerical values indicate the number of leads. Stimulation model with patient-specific tractography.

|  | **nº leads** | **1 dev** | **2 dev** | **3 dev** | **1-2 dev** |
| --- | --- | --- | --- | --- | --- |
| **All matching** | 3 |  |  |  |  |
| **Matching & extra suggestions** | 11 | 10 |  |  | 1 |
| **Matching & missing suggestions** | 12 | 12 |  |  |  |
| **None matching** | 7 | 5 | 1 |  | 1 |
| **No best clinical** | 3 |  |  |  |  |
| **Total leads** | 36 |  |  |  |  |

Supplementary Table 6. Level deviations in CST model’s suggestions compared to worst levels in the clinical monopolar review. Numerical values indicate the number of leads. Stimulation model with patient-specific tractography.

|  | **nº leads** | **1 dev** | **2 dev** | **3 dev** | **1-2 dev** | **1-2-3 dev** |
| --- | --- | --- | --- | --- | --- | --- |
| **All matching** | 15 |  |  |  |  |  |
| **Matching & extra suggestions** | 13 | 5 |  |  | 2 | 7 |
| **Matching & missing suggestions** | 3 | 3 |  |  | 1 |  |
| **None matching** | 9 | 3 | 1 | 1 | 3 | 1 |
| **Total leads** | 40 |  |  |  |  |  |

Supplementary Table 7. Level deviations in combined (HDP & CST) model’s suggestions compared to best levels in the clinical monopolar review. Numerical values indicate the number of leads. Stimulation model with patient-specific tractography.

|  | **nº leads** | **1 dev** | **2 dev** | **3 dev** | **1-2 dev** |
| --- | --- | --- | --- | --- | --- |
| **All matching** | 4 |  |  |  |  |
| **Matching & extra suggestions** | 12 | 11 |  |  | 1 |
| **Matching & missing suggestions** | 10 | 9 |  |  | 1 |
| **None matching** | 6 | 5 | 1 |  | 1 |
| **No best clinical** | 2 |  |  |  |  |
| **No best model** | 2 |  |  |  |  |
| **Total leads** | 36 |  |  |  |  |

Supplementary Table 8. Contact deviations in HDP model’s suggestions compared to best contacts in the clinical monopolar review. Numerical values indicate the number of leads. Stimulation model with patient-specific tractography.

|  | **nº leads** | **Same level** | **Different level** |
| --- | --- | --- | --- |
| **All matching** | 7 |  |  |
| **Matching & extra suggestions** | 6 | 3 | 3 |
| **Matching & missing suggestions** | 2 |  | 2 |
| **None matching**  **(stimulation threshold diff.)** | 19 | 9  (0.778 mA) | 10 |
| **Total leads** | 37 |  |  |

Supplementary Table 9. Contact deviations in CST model’s suggestions compared to worst contacts in the clinical monopolar review. Numerical values indicate the number of leads. Stimulation model with patient-specific tractography.

|  | **nº leads** | **Same level** | **Different level** |
| --- | --- | --- | --- |
| **All matching** | 14 |  |  |
| **Matching & extra suggestions** | 8 | 1 | 7 |
| **Matching & missing suggestions** | 9 | 6 | 3 |
| **None matching**  **(stimulation threshold diff.)** | 9 | 2  (1.87 mA) | 7  (1.87 mA) |
| **Total leads** | 40 |  |  |

Supplementary Table 10. Contact deviations in combined (HDP & CST) model’s suggestions compared to best contacts in the clinical monopolar review. Numerical values indicate the number of leads. Stimulation model with patient-specific tractography.

|  | **nº leads** | **Same level** | **Different level** |
| --- | --- | --- | --- |
| **All matching** | 7 |  |  |
| **Matching & extra suggestions** | 7 | 4 | 3 |
| **Matching & missing suggestions** | 1 |  | 1 |
| **None matching**  **(stimulation threshold diff.)** | 18 | 9  (0.778 mA) | 9 |
| **No best clinical** | 2 |  |  |
| **No best model** | 2 |  |  |
| **Total leads** | 37 |  |  |

Supplementary Table 11. Directional levels tested.

| **80 directional levels**  (40 leads) | 6 levels in a lead with lesion effect (3 leads) | | |
| --- | --- | --- | --- |
|  | 74 levels in leads without lesion effect | 45 tested | |
|  |  | 29 not tested | 13 levels where ring mode stimulation had side effect before effect |
|  |  |  | 16 ring mode stimulation |

Supplementary Table 12. Directional contacts tested

| **240 directional contacts**  (40 leads) | 18 contacts in leads with lesion effect | | |
| --- | --- | --- | --- |
|  | 222 contacts in leads without lesion effect | 135 tested  *(60.8%)* | |
|  |  | 87 not tested *(39.2%)* | 39 contacts where ring mode stimulation had side effect before effect *(17.6%)* |
|  |  |  | 48 ring mode stimulation (effect th. < 3mA & margin for side effect th. usually > 2 mA)  *(21.6%)* |

# Supplementary Information

**Computation time**

Computation time will highly depend on the computer used for the analysis. In this study we used a workstation (AMD® Ryzen Threadripper 2970wx 24-cores, 64 GB memory, NVIDIA Quadro RTX 4000) in which several steps could be executed in parallel. The total computation time was about 15.5 hours per patient. In the clinical practice, DBS cases are planned weeks in advance so our pipeline could be implemented. Still, it requires training and experience with the several software we used.

- Freesurfer: 4-5h for a normal subject, can vary depending on subject’s anatomy
- Preprocessing part 1 (convert images, denoising, unringing, synb0 and prepare data for eddy): 30 mins
- Preprocessing part 2 (eddy, estimate response function, obtain fiber orientation distribution, normalization): 40 mins
- Normalization warps (diffusion to structural, structural to MNI): 60 mins
- Tractography (5-tissue-type segmentation, whole-brain tractogram, SIFT2): 50 mins
- Tractography of HDP and CST (co-registration of ROIs and ROAs and selection of streamlines): 10 mins
- Lead localization: 45 mins
- VTA generation: 7.5 hours per patient (about 1.5 mins per VTA with SimBio/Fieldtrip, 300 VTAs in total per patient)
- Pathway activation by VTAs: 5 min
- Model suggestions: 5 min
